# *Bacillus anthracis* Edema Toxin Inhibits Efferocytosis in Human Macrophages and Alters Efferocytic Receptor Signaling

**DOI:** 10.3390/ijms20051167

**Published:** 2019-03-07

**Authors:** Zijian Pan, Eric K. Dumas, Christina Lawrence, Lance Pate, Sherri Longobardi, Xiaodong Wang, Judith A. James, Susan Kovats, A. Darise Farris

**Affiliations:** 1 Arthritis and Clinical Immunology Program, Oklahoma Medical Research Foundation, Oklahoma City, OK 73104, USA; zijian-pan@omrf.org (Z.P.); eric_dumas@hms.harvard.edu (E.K.D.); christina-lawrence@omrf.org (C.L.); lcpate86@att.net (L.P.); sherri-longobardi@omrf.org (S.L.); judith-james@omrf.org (J.A.J.); susan-kovats@omrf.org (S.K.); 2 Department of Microbiology and Immunology, University of Oklahoma Health Sciences Center, Oklahoma City, OK 73104, USA; 3 Center for Integrative Chemical Biology and Drug Discovery, Division of Chemical Biology and Medicinal Chemistry, Eshelman School of Pharmacy, University of North Carolina at Chapel Hill, Chapel Hill, NC 27599, USA; xiaodonw@unc.edu; 4 Departments of Medicine and Pathology, University of Oklahoma Health Sciences Center, Oklahoma City, OK 73104, USA

**Keywords:** *Bacillus anthracis*, Edema Toxin, macrophage, efferocytosis

## Abstract

The *Bacillus anthracis* Edema Toxin (ET), composed of a Protective Antigen (PA) and the Edema Factor (EF), is a cellular adenylate cyclase that alters host responses by elevating cyclic adenosine monophosphate (cAMP) to supraphysiologic levels. However, the role of ET in systemic anthrax is unclear. Efferocytosis is a cAMP-sensitive, anti-inflammatory process of apoptotic cell engulfment, the inhibition of which may promote sepsis in systemic anthrax. Here, we tested the hypothesis that ET inhibits efferocytosis by primary human macrophages and evaluated the mechanisms of altered efferocytic signaling. ET, but not PA or EF alone, inhibited the efferocytosis of early apoptotic neutrophils (PMN) by primary human M2 macrophages (polarized with IL-4, IL-10, and/or dexamethasone) at concentrations relevant to those encountered in systemic infection. ET inhibited Protein S- and MFGE8-dependent efferocytosis initiated by signaling through MerTK and αVβ5 receptors, respectively. ET inhibited Rac1 activation as well as the phosphorylation of Rac1 and key activating sites of calcium calmodulin-dependent kinases CamK1α, CamK4, and vasodilator-stimulated phosphoprotein, that were induced by the exposure of M2(Dex) macrophages to Protein S-opsonized apoptotic PMN. These results show that ET impairs macrophage efferocytosis and alters efferocytic receptor signaling.

## 1. Introduction

Humans acquire anthrax by exposure to *Bacillus anthracis* spores through cutaneous, gastrointestinal, inhalational, or blood-borne routes. High levels of circulating bacteria occur in systemic anthrax [1]. Baboons infused intravenously with vegetative *B. anthracis* bacilli mimic the systemic disease as exhibited by key features of sepsis [2,3], a life threatening, dysregulated immune response to infection that results in organ failure and often leads to death. Bacterial sepsis is associated with high levels of lymphocyte apoptosis [4,5] and increased levels of circulating nucleosomes [6,7] that may arise from uncleared apoptotic cells that have become secondarily necrotic [8]. Nucleosomes contribute to acute septic pathology by promoting intra-alveolar hemorrhage, macro- and microvascular thrombosis, and organ dysfunction [9].

Lymphoid organ macrophages are responsible for the clearance of sudden increases in apoptotic cells by a process known as efferocytosis [10,11]. The inhibition of efferocytosis in macrophages may exacerbate sepsis by increasing the burden of sepsis-promoting histones and other damage-associated molecular patterns secondary to defective apoptotic cell clearance. Efferocytosis has been reported to be inhibited by elevated cellular cAMP [12] and requires the binding of macrophages to apoptotic cells followed by macrophage signaling events that lead to Rac1-dependent apoptotic cell engulfment [13,14,15]. Direct binding is mediated by tethering receptors, while indirect binding occurs via soluble proteins that bridge the binding of apoptotic cells to macrophages. There are approximately 12 known signaling receptors that can be divided into (i) those that require bridge proteins to bind apoptotic cells and (ii) those that do not [13]. Among the former, which were evaluated in this study, Tyro3, Axl, and MerTK (TAM family) require the bridge proteins Gas6 or Protein S [16], while αVβ3 and αVβ5 require MFGE8 [17,18] or CCN1 [19]. Efferocytic macrophages in secondary lymphoid organs express MerTK [10] and alternative/M2-like markers CD163 and CD206 [20]. Glucocorticoids such as dexamethasone (Dex), which have been historically used to treat severe sepsis [21,22], enhance macrophage efferocytosis by increasing the expression of the efferocytosis receptor MerTK and its cofactors Protein S and Gas6 [23,24].

In addition to its poly-d-glutamic acid capsule, the major known *B. anthracis* virulence factors include Lethal Toxin (LT) and Edema Toxin (ET), formed by the association of the cell-binding protein Protective Antigen (PA) with the active components Lethal Factor (LF) or Edema Factor (EF), respectively [25,26]. PA binds to at least two independent receptors on the target cells [27,28], undergoes cleavage and multimerization on the cell surface, and then facilitates the binding and translocation of the LF/EF moieties into the cytosol, where they exert their toxic activities. EF is a calcium- and calmodulin-dependent adenylate cyclase that increases intracellular cAMP concentrations to supraphysiologic levels [29]. Mechanisms of ET-induced virulence and tissue damage during infection are not fully understood but may involve the inhibition of innate immunity during early stage infection and direct effects on liver tissue [26]. ET has numerous effects on immune cells, such as the inhibition of macrophage chemotaxis [30] and phagocytosis [31], the rescue of macrophages from Toll-like receptor 4-induced apoptosis [32], the inhibition of neutrophil priming and motility [33,34,35], the alteration of dendritic cell cytokine secretion, maturation and chemotaxis [36,37,38], the suppression of T cell activation and chemotaxis [30,39,40], and the skewing of CD4^+^ T cell differentiation to the Th2 subset [41].

As efferocytosis is sensitive to cAMP [12], this study tested the hypothesis that ET inhibits efferocytosis initiated by MerTK and integrin αVβ5 signaling pathways and explored the intracellular signaling events impacted. The results demonstrate that ET inhibits macrophage-mediated efferocytosis, Rac1 signaling, and the phosphorylation of Ca^2+^/calmodulin-dependent protein kinases, Rac1 and vasodilator-stimulated phosphoprotein (VASP) induced by apoptotic cell exposure.

## 2. Results

### 2.1. Bacillus anthracis Edema Toxin (ET) Inhibits Efferocytosis in a Dose-Dependent Manner

**Figure 1:**
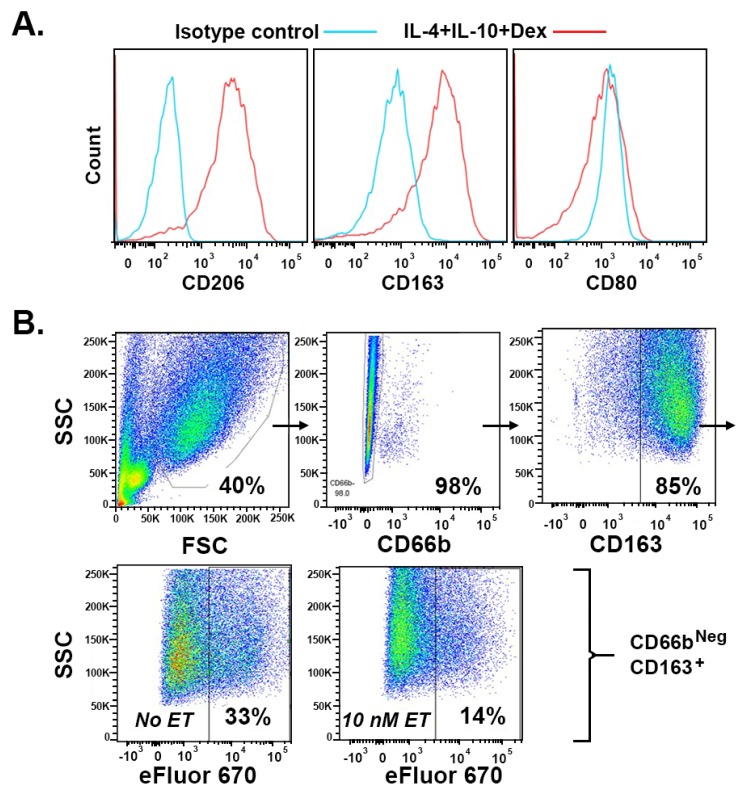
M2(IL-4+IL-10+Dex) macrophages express M2 markers and are more efferocytic in the absence of ET. (**A**) M2(IL-4+IL-10+Dex) macrophages express CD206 and CD163 but not CD80; (**B**) The representative gating of efferocytic M2(IL-4+IL-10+Dex) macrophages that were pretreated in the absence (No ET) or presence (10 nM ET) of ET: After excluding un-engulfed apoptotic polymorphonuclear neutrophil (PMN) (FSC vs. SSC, upper left) and macrophages that have surface bound apoptotic PMN detected with the PMN-specific marker CD66b (upper center), the M2 macrophages were selected by positivity for CD163 (upper right). The efferocytic cells were identified as CD163^+^CD66b^−^ macrophages that had internalized eFluor670-labeled apoptotic PMN. The data are representative of five independent experiments showing similar results.

**Figure 2:**
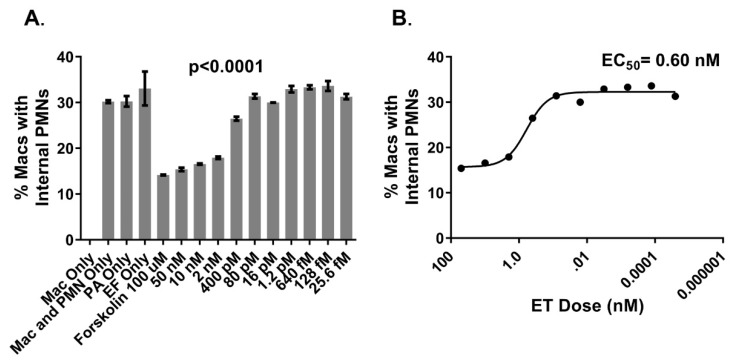
ET inhibits efferocytosis by M2(IL-4+IL-10+Dex) macrophages at a 50% effective concentration (EC_50_) of 0.6 nM: (**A**) The inhibition of efferocytosis of apoptotic PMNs by various doses of ET. The controls included macrophages without added apoptotic PMN or ET (Mac Only), macrophages and apoptotic PMN without ET components (Mac and PMN Only), macrophages and apoptotic PMN with PA but without EF (PA Only), macrophages and apoptotic PMN with EF but without PA (EF Only), and an alternative stimulus for the induction of cAMP (Forskolin 100 μM). The data were analyzed by one-way ANOVA compared to wells lacking ET; (**B**) The calculated EC_50_ of the efferocytosis inhibition using log-transformed ET dose and four-parameter nonlinear regression.

Macrophages in secondary lymphoid organs are likely to be important for clearing apoptotic lymphocytes in sepsis and for expressing M2-associated markers CD163 and CD206 [20]. As M2 polarizing stimuli are known to promote pro-efferocytic macrophage phenotypes [23,24,42] and the expression of CD163 and CD206 [43,44], we assessed whether ET could inhibit efferocytosis by human macrophages polarized with a combination of the M2 stimuli IL-4, IL-10, and dexamethasone. M2(IL-4+IL-10+Dex) macrophages expressed high levels of CD206 and CD163 but did not upregulate CD80, a marker characteristic of classically activated/M1-like macrophages [45] (Figure 1A). In a representative experiment, 30% of the CD163^+^ M2(IL-4+IL-10+Dex) macrophages had engulfed eFluor670-labeled apoptotic PMN after 2 h of coculture, whereas pretreatment of the macrophages with ET for 4 h substantially impaired efferocytosis (Figure 1B). The pretreatment of M2(IL-4+IL-10+Dex) macrophages with various concentrations of ET resulted in the dose-dependent inhibition of efferocytosis, with a calculated 50% effective concentration (EC_50_) of 0.6 nM (Figure 2).

### 2.2. Efferocytosis by Monocyte-Derived Macrophages Polarized by Dexamethasone (Dex) Depends on Protein S in the Absence of Serum

**Figure 3:**
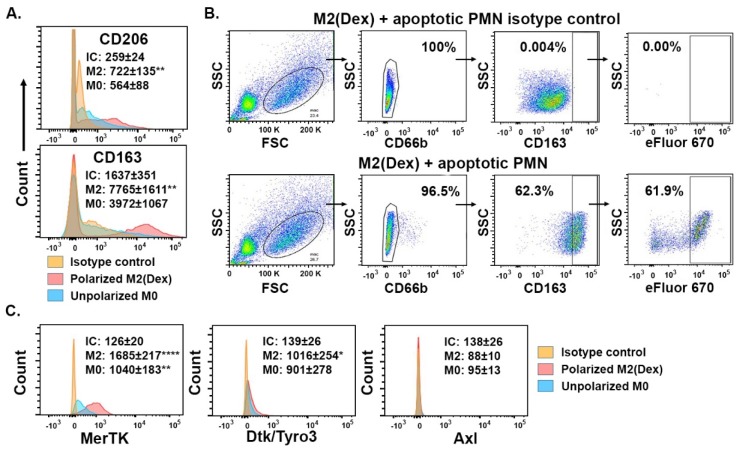
Monocyte-derived macrophages polarized by dexamethasone (M2(Dex) macrophages) expressed M2 markers and were highly efferocytic. (**A**) The histogram overlays showing the cell surface expression of CD206 and CD163 surface markers on M2(Dex) macrophages (red, M2), unpolarized M0 macrophages (blue, M0), or isotype control antibodies binding to M2(Dex) macrophages (light orange, IC). The mean fluorescence intensities (MFI) ± SEM of 10 independent donors are indicated; (**B**) The gating of efferocytosis by M2(Dex) macrophages shows (left to right) the successive exclusion of un-engulfed apoptotic PMN (FSC vs. SSC gate), the exclusion of macrophage cell surface bound CD66b^+^ PMN, the inclusion of CD163^+^ M2(Dex) macrophages, and the percentage of CD163^+^CD66b^−^ macrophages containing engulfed eFluor670^+^ PMN. The lower panels show efferocytosis gating. The upper panels depict the staining of cells from the same experiment with the isotype control antibodies for CD66b and CD163; (**C**) M2(Dex) macrophages express MerTK and Tyro3 but not Axl TAM family members. MFI ± SEM of 10 independent donors are indicated. * *p* < 0.05, ** *p* < 0.01, and **** *p* < 0.0001 by one-way ANOVA with Tukey’s multiple comparison posttest compared to IC.

Dexamethasone (Dex) alone has been shown to enhance macrophage efferocytosis by a Protein S- and MerTK-dependent mechanism [23], as well as to increase the macrophage expression of CD163 [44] and CD206 [43]. To create a more defined system to assess the effect of ET on MerTK-dependent efferocytosis, we first sought to confirm that Dex consistently induces these phenotypes in human monocyte-derived macrophages by evaluating its effect on human macrophage cell surface markers and efferocytic function using flow cytometry. As expected, macrophages polarized overnight by Dex (M2(Dex) macrophages) expressed increased levels of M2-related surface markers CD206 and CD163 (Figure 3A) and were highly efferocytic, with over 60% of macrophages engulfing the labeled apoptotic PMN after 1 h of coculture (Figure 3B). The M2(Dex) macrophages expressed elevated surface levels of MerTK compared to the macrophages incubated overnight in the absence of Dex (Figure 3C). In contrast, the expression of Tyro3 or Axl TAM family members were not upregulated on the M2(Dex) macrophages (Figure 3C).

**Figure 4:**
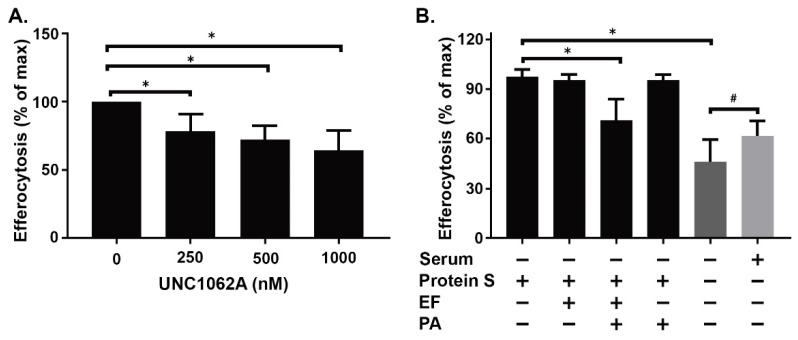
Serum-dependent efferocytosis by M2(Dex) macrophages signals through MerTK and is inhibited by ET. (**A**) The efferocytosis by M2(Dex) macrophages was preincubated for 1 h with various concentrations of the selective MerTK inhibitor UNC1062A and then incubated with apoptotic human PMN in the presence of serum for 1 h; (**B**) The efferocytosis by M2(Dex) macrophages waws preincubated with EF, PA, or ET (PA+EF) or left untreated for 4 h and then incubated with Protein S- or serum-opsonized apoptotic human PMN for 1 h. The percentages of efferocytic CD206^+^ M2(Dex) macrophages are reported. The data are presented as mean ± SEM of six individual donors and analyzed using one-way ANOVA followed by Dunnet’s multiple comparisons posttest (* *p* < 0.05) or Student’s *t*-test (# *p* < 0.05). The representative flow cytometry gatings for Figure 4A,B are shown in Figures S2 and S3, respectively.

To establish the involvement of MerTK in efferocytosis by M2(Dex) macrophages in our hands, the polarized cells were pretreated with various concentrations of a selective MerTK inhibitor (UNC1062A) [46] for 1 h prior to coculture with apoptotic human PMN and to the assessment of efferocytosis by flow cytometry. UNC1062A suppressed efferocytosis by human M2(Dex) macrophages in a dose-dependent manner (Figure 4A), indicating that efferocytosis by these macrophages is dependent on MerTK in our system. Moreover, UNC1062A did not induce intracellular cAMP in M2(Dex) macrophages (Figure S1A), supporting its specific effect on MerTK signaling. To determine if ET inhibits efferocytosis initiated by MerTK signaling, human M2(Dex) cells were preincubated with 10 nM EF, 10 nM PA, or ET (10 nM PA + 10 nM EF) or left untreated for 4 h. MerTK-dependent efferocytosis was assessed by measuring the engulfment of Protein S-opsonized human PMN in the absence of serum by flow cytometry. The opsonization of apoptotic PMN with both serum and Protein S enhanced efferocytosis by M2(Dex) macrophages (Figure 4B). Furthermore, ET, but not PA or EF alone, inhibited Protein S-enhanced efferocytosis by M2(Dex) macrophages (Figure 4B). The evaluation of intracellular cAMP in M2(Dex) macrophages treated with EF, PA, or ET confirmed the upregulation of cAMP by ET (Figure S1B).

### 2.3. MFGE8-Enhanced Efferocytosis by Human M2(Dex) Macrophages Is Associated with Integrin αVβ5 Expression and Inhibited by Edema Toxin (ET)

**Figure 5:**
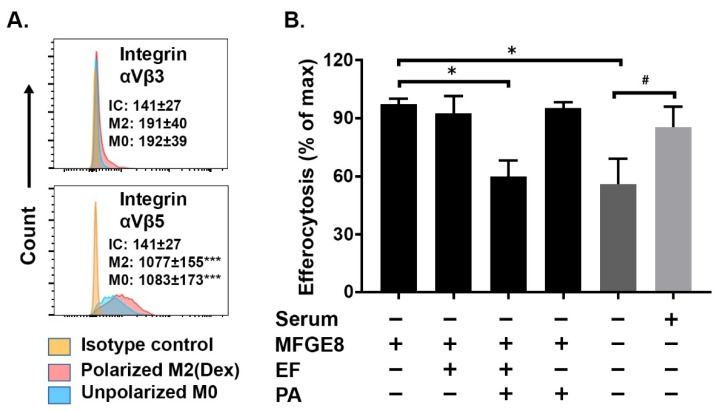
The edema toxin (ET) inhibition of MFGE8-enhanced efferocytosis by human M2(Dex) macrophages is associated with integrin αVβ5. (**A**) The histograms show the expression of cell surface αVβ5 but not αVβ3 integrin on M2(Dex) macrophages (red, M2) and unpolarized M0 macrophages (blue, M0) compared to M2(Dex) macrophages stained with isotype control antibodies (orange, IC). The mean fluorescence intensities (MFI) ± SEM of 10 independent donors are indicated. *** *p* < 0.001 by one-way ANOVA with Tukey’s multiple comparison posttest compared to IC; (**B**) M2(Dex) macrophages were preincubated with EF, PA, or ET (PA+EF) or left untreated for 4 h and then incubated with apoptotic human PMN opsonized with MFGE8 or serum. The percentages of efferocytic CD163^+^ M2(Dex) macrophages determined by flow cytometry are reported. The data presented are the mean ± SEM of four individual donors and analyzed using one-way ANOVA followed by Dunnet’s multiple comparisons test (* *p* < 0.05) or Student’s *t*-test (# *p* < 0.05).

Integrins αVβ3 and αVβ5 can act as efferocytic signaling receptors and can depend on soluble cofactor(s) present in the serum such as MFGE8 [17,18]. As efferocytosis by M2(Dex) macrophages was increased in the presence of serum (Figure 4B), we asked whether efferocytosis by M2(Dex) macrophages may be promoted by αV integrins. We first assessed the expression of αVβ3 and αVβ5 on the macrophages using flow cytometry. As shown in Figure 5A, M2(Dex) macrophages expressed integrin αVβ5 but not integrin αVβ3. Efferocytosis by M2(Dex) macrophages in serum-free conditions was significantly enhanced by the opsonization of apoptotic PMN with recombinant MFGE8 (Figure 5B), indicating that efferocytosis by these macrophages depends on αVβ5 in addition to MerTK. To determine if ET inhibits efferocytosis under MFGE8/αVβ5-dependent conditions, M2(Dex) macrophages were preincubated with 10 nM EF, 10 nM PA, or ET (10 nM PA + 10 nM EF) or left untreated for 4 h and then cocultured with human apoptotic PMN opsonized with MFGE8. Efferocytosis after 1 h of coculture was assessed by flow cytometry. Both serum and MFGE8 enhanced efferocytosis by M2(Dex) macrophages, and this MFGE8-enhanced efferocytosis was inhibited by ET but not by PA or EF alone (Figure 5B). Thus, M2(Dex) macrophages can efferocytose apoptotic PMN through an MFGE8/αVβ5-dependent process that is inhibited by ET.

### 2.4. Edema Toxin (ET) Inhibits Rac1 Activation and Alters Cytoskeletal Phosphoprotein Signaling in M2(Dex) Macrophages Induced by Protein S-Opsonized Apoptotic PMN

**Figure 6:**
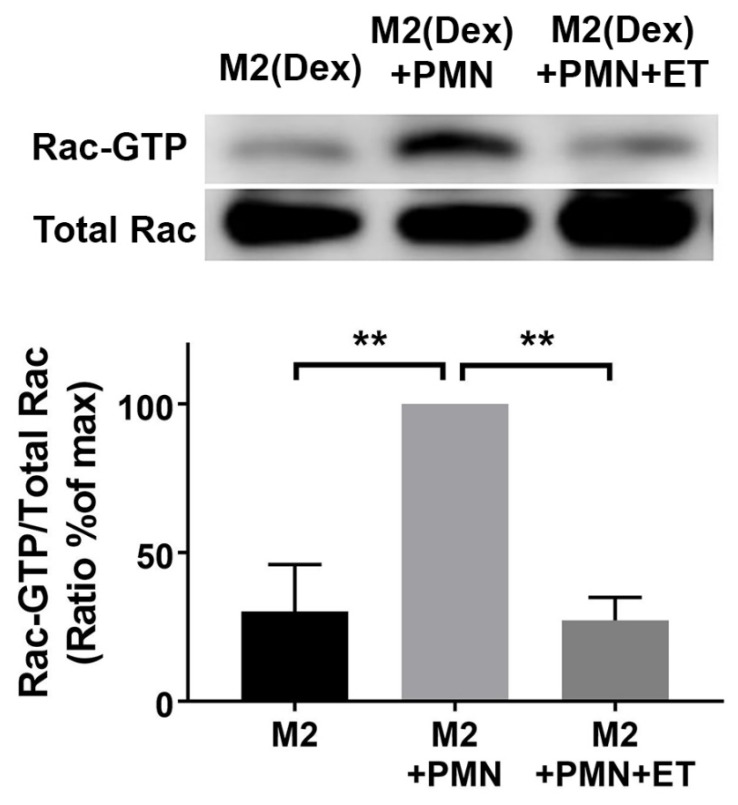
The inhibition of Rac activation by the Edema Toxin: The M2(Dex) macrophages (M2) were preincubated with or without 10 nM ET for 4 h and then incubated with human apoptotic PMNs that had been opsonized with Protein S for 15 min. The total Rac and Rac-GTP were measured from the lysates by a Western blot. Upper panel: The representative experiment shows the active and total Rac following ECL detection of the Western blot. Lower panel: The quantification of active Rac is expressed as the mean ± SEM of Rac-GTP/Total Rac ratios expressed as a percentage of the maximum. The data were analyzed by one-way ANOVA with Dunnet’s multiple comparison posttest (** *p* < 0.01). Treatment with ET inhibited the Rac activation in the presence of Protein S-opsonized apoptotic PMN. The data include results from four independent donors.

MerTK and αVβ5 signaling lead to Rac1 activation [47], which is essential for efferocytosis [48]. To determine if ET inhibits Rac1 activation in M2(Dex) macrophages downstream of MerTK, M2(Dex) macrophages were preincubated in the presence or absence of ET for 4 h and then cocultured with Protein S-opsonized apoptotic PMN under serum-free conditions for 15 min. Lysates from each condition were subjected to the pull-down of the active form of Rac. The total and pulled-down Rac1-GTP were quantified by a Western blot. The amount of active Rac1 pulled down from lysates of M2(Dex) macrophages that had been exposed to ET was substantially reduced (Figure 6), indicating that ET inhibits Rac1 activation downstream of the MerTK efferocytic receptor signaling. Similar results were observed in M2(Dex) macrophages stimulated with MFGE8-opsonized apoptotic PMN (Figure S4).

To explore whether ET may inhibit other signaling events that impact cytoskeletal reorganization downstream of MerTK, M2(Dex) macrophages were preincubated with ET or left untreated for 4 h and then cocultured with Protein S-opsonized apoptotic PMN as described above. After 20 min of coculture, the lysates prepared from each condition were biotinylated and assessed for binding to a phospho-proteome array in which solid-phase monoclonal antibodies capture the total or phosphorylated forms of various cytoskeletal-signaling phosphoproteins present in the biotinylated lysates. The quantification of the captured proteins, expressed as ratios of phosphorylated to total protein forms, revealed that ET significantly and consistently inhibited the phosphorylation of several phosphoproteins across three independent donors (Table 1). These included CamK1α (Thr 177), Rac1 (Ser 71), CamK4 (Thr 200), VASP (Ser 157), and MKK3 (Thr 222). In addition, ET showed a tendency to inhibit the phosphorylation of focal adhesion kinase (FAK) at Tyr 925 and VASP at Ser 238 (Table 1). Conversely, ET modestly enhanced the phosphorylation of PIP5K at Ser 307 (Table 1). In terms of the magnitude of inhibition in ET-treated M2(Dex) macrophages, CamK1α, Rac1, CamK4, and VASP were the most affected, with reductions in phosphorylation ranging from approx. 30–50% (Table 1).

**Figure 7:**
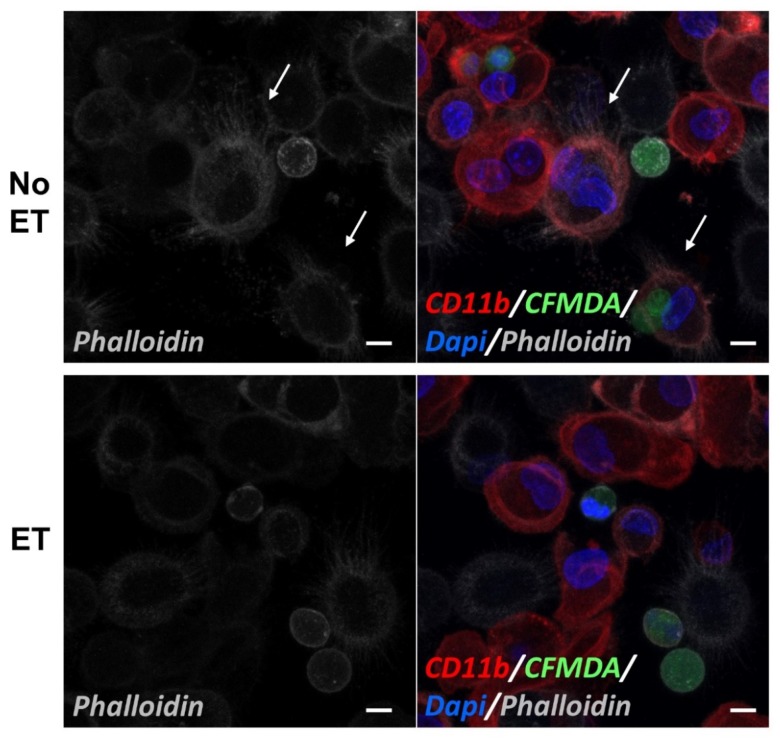
The lack of filopodia in ET-treated CD11b^+^ M2(Dex) macrophages in the presence of Protein S-opsonized apoptotic PMN. M2(Dex) macrophages plated in chambered coverglass dishes were preincubated with or without 10 nM ET for 4 h and then incubated with CFMDA-labeled, Protein S-opsonized apoptotic PMN (green) for 30 min. Following the staining for CD11b with an Alexa647-labeled monoclonal antibody to detect macrophages (red), the fixed cells were stained with Alexa568-labeled Phalloidin to detect filamentous actin (gray) and with DAPI to detect cell nuclei (blue). The z-stack images collected by confocal microscopy are presented as maximal intensity projections to permit the visualization of full cell thicknesses. In the absence of ET exposure (upper panels), Phalloidin^+^ filopodia were detected on CD11b^+^ macrophages near CFMDA-labeled apoptotic cells (white arrows). In contrast, ET-treated CD11b^+^ macrophages near CFMDA-labeled apoptotic cells (lower panels) lacked obvious filopodia. White bars = 10 μm.

The phosphorylation of CamK1α Thr 177 [49], CamK4 Thr 196 [50,51], PIP5K Ser 307 [52], VASP Ser 157 [53], and MKK3 Thr222 [54] are all activating events; thus, the pretreatment of M2(Dex) macrophages with ET results in the down-modulation of the activity of these proteins or their upstream regulators in response to Protein S-opsonized apoptotic PMN. The phosphorylation of FAK Tyr 925 [55,56] and VASP Ser 238 [57] promote cytoskeletal reorganization by altering the molecular associations and/or cellular localization of these proteins. The phosphorylation of Rac1 at Ser 71 promotes filopodia formation in fibroblasts [58]. To assess the effect of ET on actin in macrophages exposed to an efferocytic stimulus, M2(Dex) macrophages were pretreated with ET, co-incubated with Protein S-opsonized apoptotic PMN for 30 min, and then assessed for filamentous actin formation using confocal microscopy. In the absence of ET, CD11b^+^ macrophages near apoptotic PMN displayed numerous Phalloidin^+^ filopodia, whereas similarly positioned macrophages that had been pretreated with ET lacked filopodia (Figure 7).

## 3. Discussion

Edema Toxin is a recognized virulence factor of *B. anthracis* that functions as a highly active calmodulin-dependent adenylate cyclase, raising the concentration of cAMP in cells to supraphysiologic levels [29]. A selective but unidentified role for ET in systemic anthrax infection was recently demonstrated by the reduced survival of macaques infected intravenously with mutant bacilli lacking LF (ET function intact) compared to macaques similarly infected with mutant bacilli lacking PA (neither toxin present) [59]. Although ET was previously shown to inhibit the phagocytosis of *B. anthracis* bacilli through altered cytoskeletal remodeling [31], defective bacterial clearance did not appear to explain the recently identified selective role of ET in late stage anthrax in the macaque intravenous infection model [59]. Moreover, the specific effects of ET on macrophage cytoskeletal remodeling have not been previously defined beyond the roles identified for both Protein Kinase A and exchange protein activated by cAMP (EPAC), which act immediately downstream of cAMP [31].

In the present study, we tested the hypothesis that ET inhibits efferocytosis, a process of apoptotic cell engulfment and clearance that depends on cytoskeletal remodeling requiring Rac1 activation [48] and that has been shown to be inhibited by cAMP [12]. As predicted, we observed that ET inhibited efferocytosis in a dose-dependent manner. The 50% effective concentration for the inhibition of efferocytosis was calculated to be 0.6 nM. Given that circulating LT levels (defined as PA63+LF) in late stage inhalational anthrax in Rhesus macaques can reach 1150–12,100 ng/mL, equivalent to 21–79 nM [60] and that the ratio of LF:EF in the circulation of lethally infected rabbits is consistently 5:1 at various time points [61], the capacity of ET to inhibit efferocytosis is within the predicted physiologic range of ET encountered during systemic anthrax.

What are the predicted pathologic consequences of impaired efferocytosis during systemic anthrax? Data from lethal human cases of inhalational anthrax and a baboon intravenous bacillus infection model are consistent with sepsis being a factor that contributes to mortality in systemic anthrax [2,3]. A key feature of human sepsis pathology is rapid and widespread lymphocyte apoptosis [5]. Failure to effectively clear apoptotic lymphocytes leads to their secondary necrosis and the subsequent release of nucleosomes/histones and other toxic damage-associated molecular patterns [62]. Circulating nucleosomes/histones cause key features of sepsis pathology, including intra-alveolar hemorrhage, macro- and microvascular thrombosis, and organ dysfunction [9]. Based on these predictions, it was important that we test the impact of ET on efferocytic macrophages having the phenotype of those residing in human secondary lymphoid organs. Efferocytic macrophages in human lymph nodes express CD163 and CD206 [10]. We showed that human monocyte-derived macrophages either expressed or could be induced to express these markers by polarization with IL-4+IL-10+Dex or Dex alone and that ET could inhibit efferocytosis of macrophages with this phenotype.

Understanding the precise mechanisms whereby ET inhibits efferocytosis could lead to new strategies to restore efferocytosis in the setting of late stage anthrax and other forms of acute sepsis. To produce therapeutic effects, however, such strategies should preserve bacterial phagocytosis and/or be used in the context of effective antibiotic therapy. Thus, to further understand how ET subverts intracellular signaling events required for the engulfment of apoptotic cells, efferocytosis assays were conducted under defined conditions. We used conditions that promoted the initiation of efferocytic signaling through MerTK, a relevant efferocytic signaling receptor in the secondary lymphoid organs [10] or through αVβ5 integrin that can signal for efferocytosis on its own [18] or in cooperation with MerTK [63]. The expression of MerTK, Tyro 3, and αVβ5 (but not Axl or αVβ3) by M2(Dex) macrophages ensured that signaling initiated by Protein S- or MFGE8-opsonized apoptotic PMN preferentially occurred through these receptors.

An evolutionarily conserved signaling pathway that leads to efferocytosis involves the recruitment of a complex of p130Cas, CrkII, and DOCK-180 proteins to the vicinity of the signaling receptor, resulting in Rac1 activation that leads to cytoskeletal reorganization and engulfment [64]. Experiments conducted in HEK-293T cells showed that MerTK activation leads to the Src-mediated phosphorylation of FAK on Tyr 861, followed by the recruitment of FAK to αVβ5, the increased phosphorylation of p130Cas, the increased formation of the p130Cas/CrkII/Dock180 complex, enhanced Rac1 activation, and efferocytosis [63]. The signaling was enhanced synergistically by the presence of αVβ5 [63]. Our studies in primary human macrophages revealed that ET could substantially and reproducibly inhibit the activation of Rac1 following the exposure of M2(Dex) macrophages to Protein S-opsonized apoptotic PMN, showing a perturbation of the efferocytic signaling pathway by ET. Although we did not observe a significant inhibition of the phosphorylation of Tyr 861 on FAK across three independent donors, a 49% and 28% reduction of phosphorylation of this residue in the presence of ET was observed in two of the three donors (not shown). We did, however, observe a modest but consistent reduction in phosphorylation of FAK Tyr 925. Notably, the phosphorylation of this residue was required for FAK-mediated cell protrusion in mouse embryonic fibroblasts that depended on the p130Cas/Dock180/Rac1 signaling pathway [56], suggesting that an interruption of the FAK activation by ET may contribute to its capacity to inhibit efferocytosis. Furthermore, the phosphorylation of FAK at Tyr 925 played a causative role in stimulating the migration of RAW 264.7 mouse macrophages [65], consistent with a role for the inhibition of FAK in the ET-mediated suppression of cell migration [30,35].

The most substantial changes in cytoskeleton signaling protein phosphorylation events downstream of MerTK that were induced by the pre-exposure of macrophages to ET included the reduced phosphorylation of Ser-71 on Rac, as well as the reduced phosphorylation of activating Thr residues of CamKs 1α and 4 and Ser residues of VASP. Though originally reported to be an inhibitory phosphorylation event, Ser-71 phosphorylation on Rac was more recently found to promote filopodia formation in fibroblasts [58]. The predicted effects downstream of Cam kinases [50,51] include altered transcription. Notably, ET has been shown to alter the transcription of macrophages as early as 3 h posttreatment, and these changes have been proposed to impact cytoskeletal remodeling [31]. VASP has not previously been implicated in efferocytosis, but its localization to the phagocytic cup was shown to be essential for actin cytoskeletal reorganization, the extension of pseudopodia, and phagocytosis by macrophages [66]. In Drosophila macrophages, Ena/VASP stimulates WAVE regulatory complex-mediated actin assembly in the presence of Rac, which is necessary for lamellipodia formation [67], an event also known to be essential for efferocytosis. Consistent with an impact of these phosphorylation events on filopodia formation, we observed a reduced filamentous actin-positive filopodia in ET-treated CD11b^+^ M2(Dex) macrophages exposed to apoptotic PMN (Figure 7). However, this observation requires confirmation in live cell imaging studies to effectively capture actin reorganization events occurring during the process of efferocytosis.

This study establishes the capacity of *B. anthracis* ET to inhibit macrophage-mediated efferocytosis at doses relevant to systemic infection and further provides novel insight into putative molecular mechanisms.

## 4. Materials and Methods

### 4.1. Isolation, Differentiation, and Polarization of Human Monocytes

The acquisition and use of all human cells in this study was compliant with the Declaration of Helsinki and was reviewed and approved by the Oklahoma Medical Research Foundation Institutional Review Board, ensuring the protection of human subjects. Fresh buffy coats (containing white blood cells and platelets) of healthy blood donors were purchased from the Oklahoma Blood Institute, USA. Mononuclear cells were isolated using Lympholyte-H Cell Separation Media (Cedarlane, Burlington, NC, USA). The monocytes were either purified using an EasySep Human Monocyte Isolation Kit (StemCell Technologies, Cambridge, MA, USA) or isolated as adherent cells according to the following procedure: isolated mononuclear cells were resuspended at 4 × 10^6^/mL in Iscove’s Modified Dulbecco’s Medium (IMDM, Gibco, Grand Island, NY, USA) and adhered to nontreated 100 mm × 20 mm tissue culture dishes (Corning, Corning, NY, USA) for 1 h at 37 °C in 5% CO_2_. Nonadherent cells were removed by washing with IMDM. To generate macrophages, monocytes were cultured for 6 or 7 days with 50 ng/mL M-CSF (Peprotech, Rocky Hill, NJ, USA) in either RPMI 1640 with 10% FBS, 2 mM glutamine, 100 U pen/strep/mL, 10 mM HEPES buffer, and 1 mM sodium pyruvate (for M2(IL-4+IL-10+Dex) macrophages) or in IMDM containing 10% FBS and 100 U pen/strep/mL (for M2(Dex) macrophages). On day 7 or 8, adherent cells were detached using a Trypsin-EDTA solution (Millipore Sigma, St. Louis, MO, USA) and resuspended in a fresh medium with 50 ng/mL M-CSF and 20 ng/mL IL-10 (Peprotech), 20 ng/mL IL-4 (Peprotech), and/or 10 nM dexamethasone (Dex) (Millipore Sigma) at 5 × 10^5^/well in temperature-sensitive 12 well-plates (Nunc UpCell, Rochester, NY, USA) overnight.

### 4.2. Generation of Early Apoptotic Human Polymorphonuclear Neutrophils (PMN)

Polymorphonuclear neutrophils (PMN) were isolated from fresh human blood using the EasySep Direct Human Neutrophil Isolation Kit (StemCell Technologies, Cambridge, MA, USA). The isolated PMN were labeled with 10 μM eFluor 670 (Cell Proliferation Dye, eBioscience, San Diego, CA, USA), and then, apoptosis was induced by UV irradiation (500 mJ/cm^2^ for 2 min and 41 s) followed by 4 h of culture at 5 × 10^6^/mL in IMDM in the absence of serum at 37 °C in 5% CO_2_ atmosphere. Cultured PMN populations were routinely >70% early apoptotic (Annexin V^+^/PI^−^) and <5% late apoptotic (Annexin V^+^/PI^+^), as determined by flow cytometry using the Annexin V Apoptosis Detection Kit FITC (eBioscience; Figure S5). Before the coculture with human M2(Dex) macrophages in various assays, apoptotic PMN were pretreated with either 12 μg/ml Protein S (Enzyme Research Laboratories, South Bend, IN, USA), 12.5 μg/mL MFGE8 (R&D Systems, Minneapolis, MN, USA), or 10% serum (FBS) for 1 h.

### 4.3. Flow Cytometry

Macrophages were detached from Nunc UpCell tissue culture plastic by incubation in cold PBS on ice for 20–30 min. After washing with ice-cold PBS containing 2% FBS, the cells (5 × 10^5^/assay) were treated with Human BD Fc block (BD Biosciences, San Jose, CA, USA) for 10 min at room temperature followed by staining with fluorophore-conjugated monoclonal antibodies for 30 min on ice. The data from washed cells were collected using an LSRII cytometer (BD Biosciences) and analyzed with FlowJo (Treestar, Ashland, OR, USA) software. Efferocytosis receptor antibodies (R&D Systems) were PE-labeled and included the following specificities and clones: Dtk/Tyro3 (clone 96201), Axl (108724), MerTK (125518), αVβ3 (23C6), or αVβ5 (P5H9). Other antibodies and clones included PE-Cy7-conjugated antihuman CD80 (BioLegend, San Diego, CA, USA, clone 2D10), PerCp-Cy5.5-conjugated antihuman CD206 (BioLegend, 15-2), APC-Cy7-conjugated antihuman CD163 (BioLegend, GHI/61), and BV-421-conjugated antihuman CD66b (BD Biosciences, G10F5).

### 4.4. Efferocytosis Assay

Efferocytosis was assessed by flow cytometry [68]. Briefly, 5 × 10^5^/well monocyte-derived human M2(IL-4+IL-10+Dex) macrophages or M2(Dex) macrophages were preincubated with 10 nM recombinant PA (List Biological Laboratories, Campbell, CA, USA), 10 nM recombinant EF (List Biological Laboratories), or various concentrations of PA + EF (ET) for 4 h at 37 °C in 5% CO_2_ atmosphere. Fluorescently-labeled apoptotic human PMN were preincubated with 10% serum, Protein S, or MFGE8 for 1 h unless otherwise noted and then added to the macrophages, resulting in a macrophage:PMN ratio of 1:5. After 1 h at 37 °C in 5% CO_2_ atmosphere, the percentage macrophages containing intracellular (eFluor 670^+^) but not surface-bound (surface CD66b^−^) PMN was assessed by flow cytometry.

### 4.5. MerTK Inhibition Efferocytosis Assay

Human M2(Dex) macrophages (5 × 10^5^/well) were preincubated with various concentrations (0–1 μM) of selective MerTK inhibitor UNC1062A [46] for 1 h at 37 °C in 5% CO_2_ atmosphere prior to the addition of fluorescently labeled, Protein S-opsonized, human apoptotic PMN. The final macrophage:PMN ratio was 1:5. After 1 h of coculture, the percentage of macrophages that had engulfed apoptotic PMN was determined by flow cytometry.

### 4.6. Rac Pull-Down Assay

M2(Dex) macrophages were washed four times with PBS and then incubated overnight in IMDM containing 10% FBS in the absence of M-CSF to reduce M-CSF receptor signaling to a basal state. Rested M2(Dex) macrophages were preincubated in the presence or absence of 10 nM ET for 4 h at 37 °C in 5% CO_2_ atmosphere, washed four times with PBS, and then cocultured (6 × 10^6^ human M2(Dex) macrophages/well) with either Protein S- or MFGE8-opsonized human apoptotic PMN at a 5:1 PMN:M2(Dex) macrophage ratio for 15–20 min. The Rac-GTP and total Rac were measured using a Rac Activation Assay Kit (NewEast Biosciences, King of Prussia, PA, USA), following the protocol described by the manufacturer. The total lysate and Rac-GTP pulled down from lysates were electrophoresed and blotted using the manufacturer’s provided anti-Rac polyclonal antibody (2 μg/mL). The immune-reactive bands were visualized using a peroxidase-conjugated goat anti-rabbit immunoglobulin G (IgG) (1:1000 dilution) antibody (WesternSure Goat Anti-Rabbit IgG (H+L), LI-COR) and an ECL Plus Western Blotting Detection System (Azure Biosystems, Dublin, CA, USA). The images were captured using an Azure cSeries C600 (Azure Biosystems) instrument using the imaging workflow for chemiluminescent applications. The quantifications were carried out using Image J software. The ratios of Rac-GTP/Total Rac were expressed as percentages of the maximum ratio within each individual donor.

### 4.7. Phosphoprotein Profiling Assay

M2(Dex) macrophages were washed four times with PBS and then incubated overnight in IMDM containing 10% FBS in the absence of M-CSF to reduce M-CSF receptor signaling to a basal state. Rested M2(Dex) macrophages were preincubated in the presence or absence of 10 nM ET for 4 h at 37 °C in 5% CO_2_ atmosphere, washed four times with PBS, and then cocultured (6 × 10^6^ human M2(Dex) macrophages) with Protein S-opsonized human apoptotic PMN at a 5:1 PMN:M2(Dex) macrophage ratio for 20 min. The lysates were biotinylated and used to probe the Cytoskeleton Phospho Antibody Array (Full Moon BioSystems, Sunnyvale, CA, USA) according to the manufacturer’s instructions. Biotinylated proteins captured by the arrays were detected using AlexaFluor 555-Streptavidin. The slides were scanned on a GenePix 4000B Microarray scanner at 532 nm, and the images were analyzed with GenePix Pro 7.3.1 software (Molecular Devices, San Jose, CA, USA). The fluorescence signal was obtained by calculating the Foreground (center of signal) to Background (adjacent to signal) Ratio (F/B) of each capture antibody location on the array. The ratios of signals detected by antibodies directed to phosphorylated versus un-phosphorylated proteins were compared in the presence and absence of ET.

### 4.8. Confocal Microscopy

M2(Dex) macrophages were washed four times with PBS, then seeded onto 8-well chambered coverglass plates (Nunc Lab-TekII Chambered Coverglass, Rochester, NY, USA, 2.5 × 10^5^ macrophages/well), and grown overnight in IMDM containing 10% FBS at 37 °C in 5% CO_2_ atmosphere. The cells were preincubated with or without 10 nM ET for 4 h and then cocultured with 5-chloromethylfluorescein diacetate (CFMDA, Molecular Probes, Life Technologies, Eugene, OR, USA)-labeled apoptotic PMN that had been opsonized with Protein S at a 5:1 PMN:M2(Dex) macrophage ratio. After 30 min of co-incubation, the cells were washed three times and blocked with 10% human AB serum on ice for 15 min. CD11b was detected by incubating with AlexaFluor647-labeled antihuman CD11b antibody (Clone ICRF44, Southern Biotech, Birmingham, AL, USA) on ice for 1 h. To visualize the actin filaments, the cells were fixed with 2% para-formaldehyde and then co-stained with Phalloidin-AlexaFluor568 conjugate (Invitrogen, Eugene, OR, USA) at room temperature for 20 min and mounted in Prolong Gold with 4′,6-diamidino-2-phenylindole (DAPI) (Life Technologies). The images were captured by optical sectioning on a Zeiss LSM-510 META laser-scanning microscope and cumulatively displayed as maximal intensity projections.

### 4.9. Statistical Analyses

The results are presented as mean ± SEM, with the number of independent experiments or donors indicated in the figure legends. The results were analyzed by one-way ANOVA followed by a multiple comparisons posttest (*p* < 0.05) or Student’s *t*-test (*p* < 0.05) as indicated in the figure legends (GraphPad, San Diego, CA, USA).

## Figures and Tables

**Table 1:** Edema Toxin-exposed M2(Dex) macrophages display the reduced phosphorylation of cytoskeletal signaling proteins in response to Protein S-opsonized apoptotic PMN ^a^.

Protein ^b^	Phospho Site	Mean ± SEM No ET	Mean ± SEM ET	*p*-Value ^c^	% Change ^d^
CamK1α	Thr 177	0.897 ± 0.043	0.533 ± 0.054	0.006	40.6 ↓
Rac1	Ser 71	2.990 ± 0.251	1.750 ± 0.056	0.009	41.5 ↓
CamK4	Thr 196/200	1.657 ± 0.102	1.160 ± 0.017	0.009	30.0 ↓
PIP5K	Ser 307	0.563 ± 0.023	0.657 ± 0.003	0.017	16.7 ↑
VASP	Ser 157	1.283 ± 0.166	0.630 ± 0.135	0.038	50.9 ↓
MKK3	Thr 222	0.960 ± 0.058	0.787 ± 0.019	0.046	18.0 ↓
FAK	Tyr 925	1.230 ± 0.086	0.987 ± 0.018	0.051	19.8 ↓
VASP	Ser 238	1.903 ± 0.133	1.267 ± 0.200	0.057	33.0 ↓

^a^ The ratios of the phosphorylated to total forms of the indicated proteins in cell lysates prepared from M2(Dex) macrophages exposed to Protein S-opsonized apoptotic PMN for 20 min are shown. The results compare M2(Dex) macrophages that were (ET) or were not (no ET) pretreated for 4 h with ET. The data represent the mean ± SEM of three independent experiments using cell lysates from three different donors; ^b^ CamK1α = calcium calmodulin dependent protein kinase 1α, Rac1 = Rac family small GTPase 1, CamK4 = calcium calmodulin dependent protein kinase 4, PIP5K = phosphoinositide kinase, FYVE-type zinc finger containing; VASP = vasodilator stimulated phosphoprotein, MKK3 = mitogen-activated protein kinase 3, FAK = focal adhesion kinase; ^c^ The *p*-Values determined by the *t*-test comparing the results of three independent experiments using M2(Dex) cells from three different donors; ^d^ % Change = |[(Mean No ET − Mean ET)/Mean No ET] × 100|. The arrows indicate an increase or decrease in phosphorylation in the presence of ET.

## Supplementary Materials

Supplementary materials can be found at https://www.mdpi.com/1422-0067/20/5/1167/s1.

## Abbreviations

cAMP	cyclic adenosine monophosphate
CamK	Ca^2+/^calmodulin-dependent protein kinase
EF	edema factor
ET	edema toxin
FAK	focal adhesion kinase
FBS	fetal bovine serum
PA	protective antigen
M2(Dex)	dexamethasone-polarized M2 macrophages
M2(IL-4+IL-10+Dex)	M2 macrophages polarized with a mixture of IL-4, IL-10, and dexamethasone
M-CSF	macrophage colony stimulating factor
MerTK	Mer proto-oncogene tyrosine kinase
MFGE8	milk fat globulin protein E8
MKK3	mitogen-activated protein kinase 3
PIP5K	phosphoinositide kinase, FYVE-type zinc finger containing
PMN	polymorphonuclear neutrophils
VASP	vasodilator-stimulated phosphoprotein
