# Supplementary material for: Bacillus anthracis Edema Toxin Inhibits Efferocytosis in Human Macrophages and Alters Efferocytic Receptor Signaling
Source: Int J Mol Sci. 2019 Mar 7;20(5):1167. doi: 10.3390/ijms20051167 (PMC6429438; doi:10.3390/ijms20051167)
Supplement: Supplementary file 1 [file ijms-20-01167-s001.pdf]

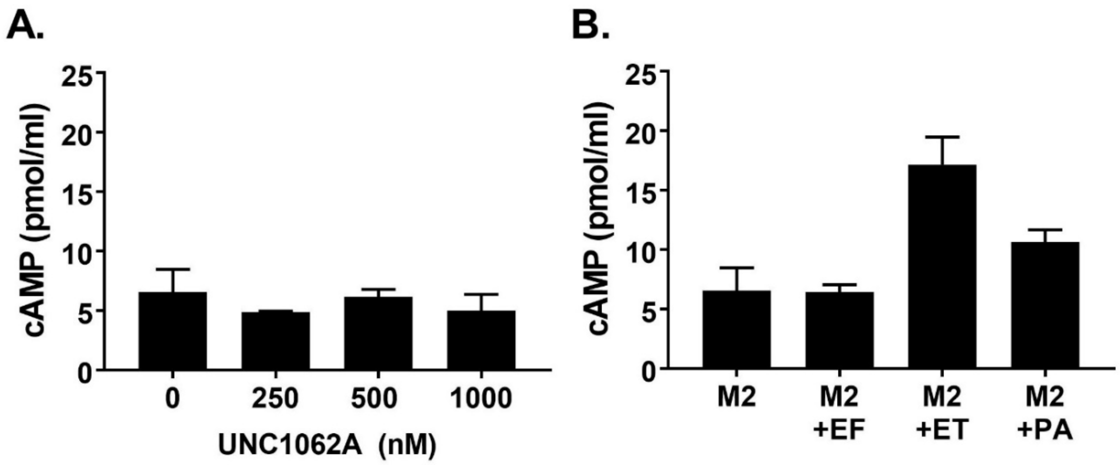

Figure S1. The intracellular cAMP levels in M2(Dex) macrophages are unaffected by the MerTK inhibitor UNC1062A and are elevated by the Edema Toxin (ET). (**A**) The macrophages were treated with the indicated concentrations of UNC1062A for 2h, washed, lysed in 0.1 M HCl, and assessed for intracellular cAMP; (**B**) The macrophages were untreated (M2) or treated with 10 nM EF, ET (10 nM each PA+EF), or 10 nM PA for 4 h; washed; lysed in 0.1 M HCl; and assessed for intracellular cAMP. The measurements used the cAMP enzyme-linked immunosorbent assay (ELISA) from Cayman Chemical, Item No. 581001, according to the manufacturer's instructions. The error bars are the SD of the averaged duplicates from two individual donors.

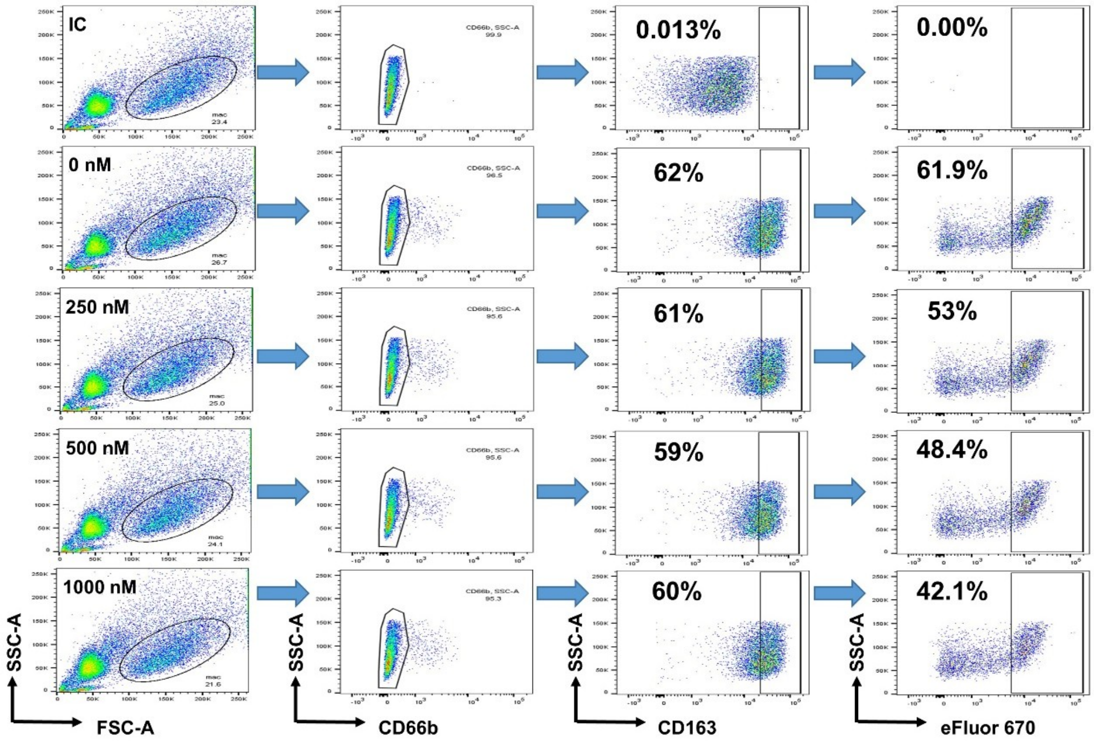

Figure S2. The representative flow cytometry gating for the experiment shown in Figure 4A: The gating of efferocytosis by M2(Dex) macrophages shows (left to right) the successive exclusion of un-engulfed apoptotic PMN (FSC vs. SSC gate), the exclusion of macrophage cell surface bound CD66b$^+$ PMN, the inclusion of CD163$^+$ M2(Dex) macrophages, and the percentage of CD163$^+$CD66b$^-$ macrophages containing engulfed eFluor670$^+$ PMN. The upper row of panels used isotype control (IC) antibodies for CD66b and CD163 to demonstrate the gating placement for positive staining in the absence of UNC1062A. The second, third, fourth, and bottom rows indicate experiments performed in the presence of various concentrations of UNC1062A as indicated. See Figure 4A for additional details.

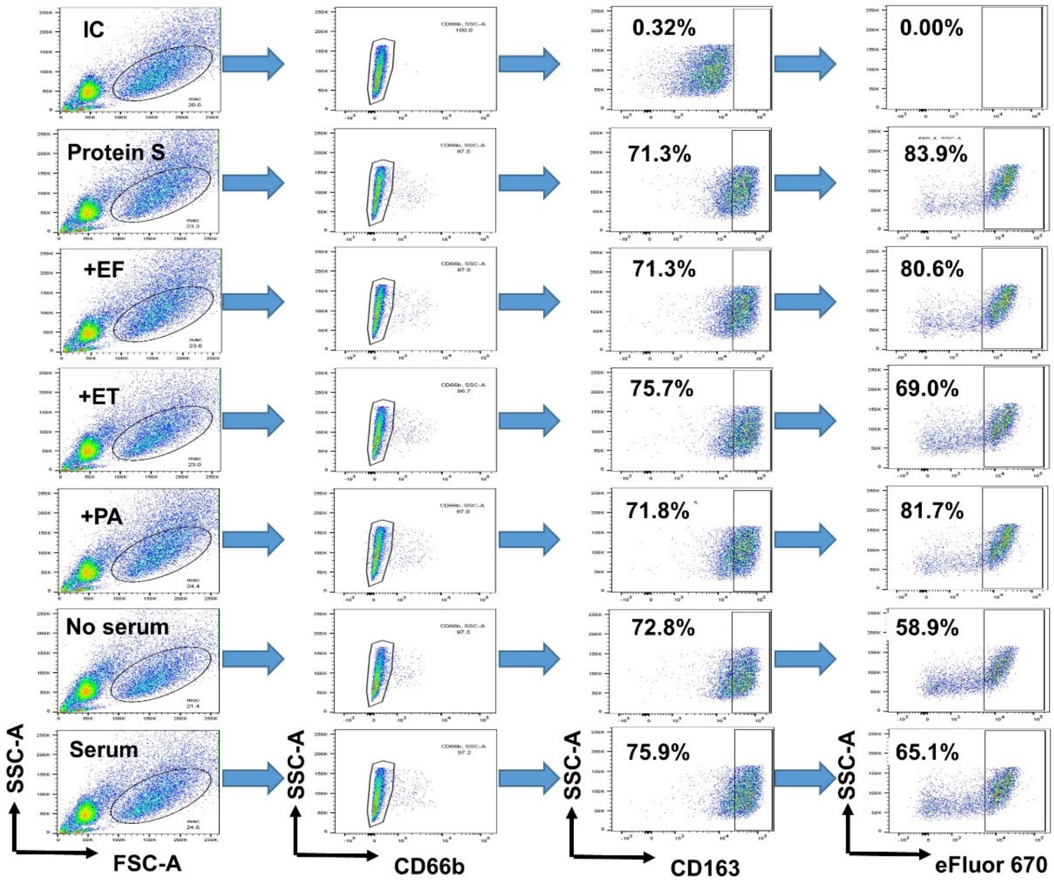

Figure S3. The representative flow cytometry gating for the experiment shown in Figure 4B: The gating of efferocytosis by M2(Dex) macrophages shows (left to right) the successive exclusion of un-engulfed apoptotic PMN (FSC vs. SSC gate), the exclusion of macrophage cell surface bound $CD66b^{+}$ PMN, the inclusion of $CD163^{+}$ M2(Dex) macrophages, and the percentage of $CD163^{+}CD66b^{-}$ macrophages containing engulfed $eFluor670^{+}$ PMN. The upper row of panels used isotype control (IC) antibodies for CD66b and CD163 in the presence of apoptotic PMN to demonstrate the gating placement for positive staining. Rows 2–5 assessed the efferocytosis of Protein S-opsonized apoptotic PMNs by M2(Dex) macrophages in the absence of PA or EF (Protein S, second row), with EF alone (+EF, third row), with PA+EF (+ET, fourth row), or with PA alone (+PA, fifth row). Rows 6 and 7 (lower two rows) used un-opsonized apoptotic PMNs (No serum) or PMNs opsonized with 10% human AB serum (Serum). See Figure 4B for further details.

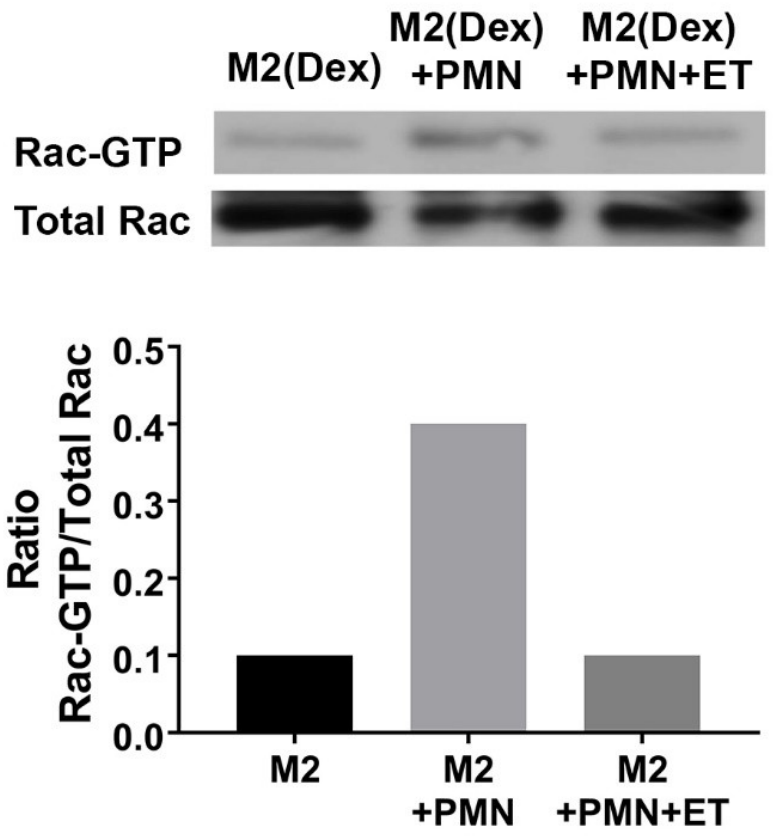

Figure S4. The inhibition of Rac activation by the Edema Toxin in response to MFGE8-opsonized apoptotic PMNs. The M2(Dex) macrophages were preincubated with or without 10 nM ET for 4 h and then incubated with human apoptotic PMNs that had been opsonized with MFGE8 for 15 min. The total Rac and Rac-GTP were measured from the lysates by a Western blot. Upper panel: The representative blot shows the active and total Rac following ECL detection of a Western blot. Lower panel: The quantification of active Rac is expressed as a ratio of Rac-GTP/Total Rac.

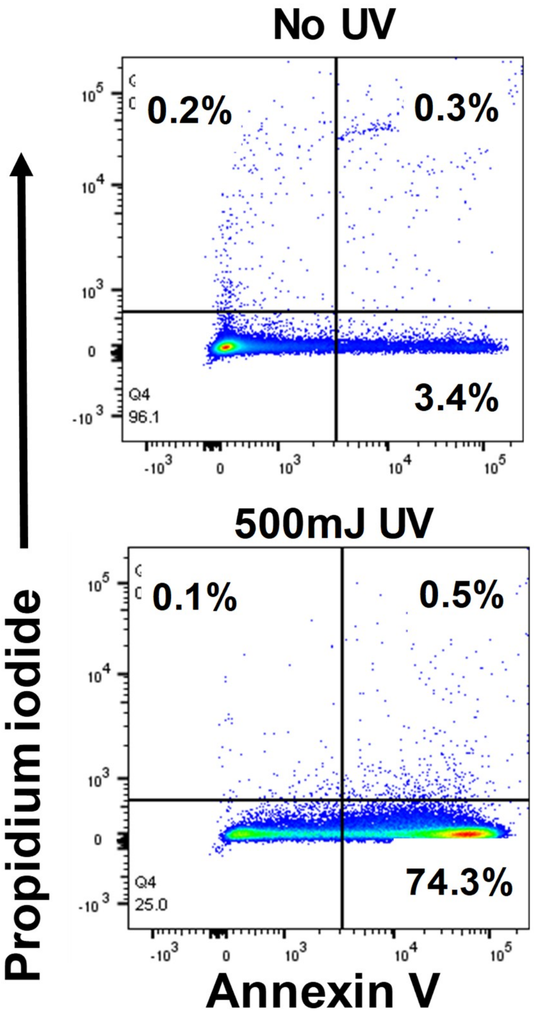

Figure S5. The representative phenotype of early apoptotic human PMN induced by UV irradiation.
